# Kinking and Torsion Can Significantly Improve the Efficiency of Valveless Pumping in Periodically Compressed Tubular Conduits. Implications for Understanding of the Form-Function Relationship of Embryonic Heart Tubes

**DOI:** 10.3390/jcdd4040019

**Published:** 2017-11-19

**Authors:** Florian Hiermeier, Jörg Männer

**Affiliations:** Group Cardio-Embryology, Institute of Anatomy and Embryology, UMG, Georg-August-University Goettingen, D-37075 Goettingen, Germany; florian.hiermeier@stud.uni-goettingen.de

**Keywords:** heart looping, blood vessel kinking, valveless pumping, Liebau effect, form-function relationship

## Abstract

Valveless pumping phenomena (peristalsis, Liebau-effect) can generate unidirectional fluid flow in periodically compressed tubular conduits. Early embryonic hearts are tubular conduits acting as valveless pumps. It is unclear whether such hearts work as peristaltic or Liebau-effect pumps. During the initial phase of its pumping activity, the originally straight embryonic heart is subjected to deforming forces that produce bending, twisting, kinking, and coiling. This deformation process is called cardiac looping. Its function is traditionally seen as generating a configuration needed for establishment of correct alignments of pulmonary and systemic flow pathways in the mature heart of lung-breathing vertebrates. This idea conflicts with the fact that cardiac looping occurs in all vertebrates, including gill-breathing fishes. We speculate that looping morphogenesis may improve the efficiency of valveless pumping. To test the physical plausibility of this hypothesis, we analyzed the pumping performance of a Liebau-effect pump in straight and looped (kinked) configurations. Compared to the straight configuration, the looped configuration significantly improved the pumping performance of our pump. This shows that looping can improve the efficiency of valveless pumping driven by the Liebau-effect. Further studies are needed to clarify whether this finding may have implications for understanding of the form-function relationship of embryonic hearts.

## 1. Introduction

In the internal convective transport systems of animals, gas or fluid flow is generated by structural elements called “biological pumps” [1,2]. In the mature cardiovascular systems of vertebrates such pumps usually possess building elements facilitating passage of the moving fluid in only one direction while blocking flow in the opposite direction. These building elements—called valves—form the structural basis for the unidirectional fluid flow normally found in the cardiovascular and lymphatic systems of adult vertebrates [3]. Irrespective of the physical mechanism working in a given biological pump, any pump action that generates unidirectional fluid flow due to the presence of valves may be classified as “valve-supported pumping”.

Valve-supported pumping seems to be the predominant way for moving fluids in a one-way fashion through the conduits of the mature cardiovascular systems of vertebrates and several invertebrates. It is, however, not the only physical mechanism for the generation of unidirectional fluid flow in animals. In the gastrointestinal and urogenital systems, for example, one-way flow is generated in the absence of valves by a mechanism called “propulsive peristalsis” [4]. For physicians, propulsive peristalsis may be the best-known representative of a group of physically diverse pumping mechanisms, which have the common feature that they all generate unidirectional fluid flow in the absence of valves. Such pumping mechanisms have been classified as “valveless pumping” [1,2,5,6,7]. Besides propulsive peristalsis, the biologically most important representative of valveless pumping may be the so-called “Liebau effect”, which is a valveless pumping phenomenon that has been uncovered by the German cardiologist Gerhart Liebau in the early 1950s [5,8,9,10,11,12].

The heart is one of the first biological pumps to form and function in vertebrate embryos. In human beings, for example, it starts beating at the beginning of the fourth week of development [13,14], which is only seven days after the first missed menstrual bleeding. During the early stages of embryonic development, the morphology and pumping action of vertebrate hearts differs considerably from that at maturity. The morphology of mature vertebrate hearts is characterized by the presence of chambers and valve-bearing segments [15]. These hearts fulfill their pumping action in a valve-supported manner. In contrast, cardiac morphology at early embryonic stages is neither characterized by the presence of chambers nor by the presence of valve-bearing segments. The early embryonic heart of vertebrates is a valveless, tubular structure whose architecture has been characterized as that of a pulsatile blood vessel [7,16,17,18,19] and its mode of action has been characterized as valveless pumping [6,8,20]. For more than 200 years, it was thought that propulsive peristalsis was the mode of valveless pumping used by the tubular embryonic heart [7]. At the present time, however, it is an open question whether the embryonic heart tube generates unidirectional blood flow via propulsive peristalsis or rather via the above-mentioned Liebau effect [7,20,21,22,23,24,25,26,27,28,29].

When comparing the morphology of tubular embryonic hearts with that of tubular blood vessels of adult vertebrates, it may become apparent, at first, that the former does not have a stable three-dimensional configuration over time, but normally undergoes remarkable morphological changes during a short time period before it starts the transformation of its tubular structure into a bi-chambered (fishes), three-chambered (lung fishes, amphibia, reptiles) or four-chambered (crocodiles, birds, mammals) pump with valve-bearing elements. The initial form of the embryonic vertebrate heart is that of a short and straight tube. This tubular blood vessel lies within a body cavity—called the primitive pericardial cavity—where it is aligned along the future cranio-caudal body axis. It has an inlet at its caudal end, with which it is connected to the venous branch of the circulation, and an outlet at its cranial end, with which it is connected to the arterial branch of the circulation. The initially short heart tube becomes elongated by continuous addition of new material to its cranial and caudal ends [30]. Due to the fact that the cranio-caudal length of the primitive pericardial cavity does not change significantly during the phase of elongation of the embryonic heart tube, the latter is mechanically forced to change its three-dimensional configuration from a straight blood vessel into a looped blood vessel [31]. Consequently, this morphogenetic process is called cardiac looping [30,32,33]. During this process, the embryonic heart tube is subjected to deforming forces that produce bending, twisting, kinking, and helical coiling of this specialized blood vessel [30,32,33,34,35,36,37,38,39].

**Figure 1:**
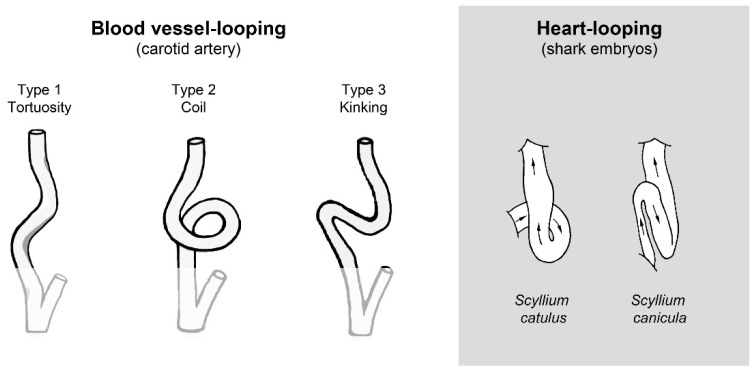
Schematic drawing illustrating the similarities between the morphological phenotypes of blood vessel looping in adult human beings and heart tube looping in vertebrate embryos. Pictures are based on arterial phenotypes as classified by Weibel and Fields [40] and on Figure 15 from Tschermak [37].

The phenomenon of blood vessel looping is well known, not only to those studying the morphogenesis and physiology of the central pulsating blood vessel of the early embryonic circulation, but also to those working on the mature cardiovascular system [40,41]. A comparison of the morphological phenotypes resulting from carotid artery looping with those produced by cardiac looping discloses striking morphological similarities between the looped configurations of the two different blood vessels (Figure 1). With respect to the mature macrovasculature, it is unclear whether such vascular deformations should be regarded as anomalous form variants, which do not significantly compromise circulation, or whether they represent pathological deformations, which may have detrimental effects on the perfusion of the organs supplied by the affected vessel [42,43]. Kinking of the carotid and iliac arteries, for example, is suspected to cause transitory ischemia of the brain or leg, respectively [44,45], and coronary artery kinking is related to cardiac death [46,47]. Surgical animal models have indeed shown that carotid artery kinking can significantly reduce the flow rate through this vessel [48]. A flow reducing effect was also observed after twisting of blood vessels [49]. Blood vessel looping is not confined to large blood vessels. It is also observed in the microvasculature, where it is suspected to exert deleterious effects on tissue perfusion [50,51].

**Figure 2:**
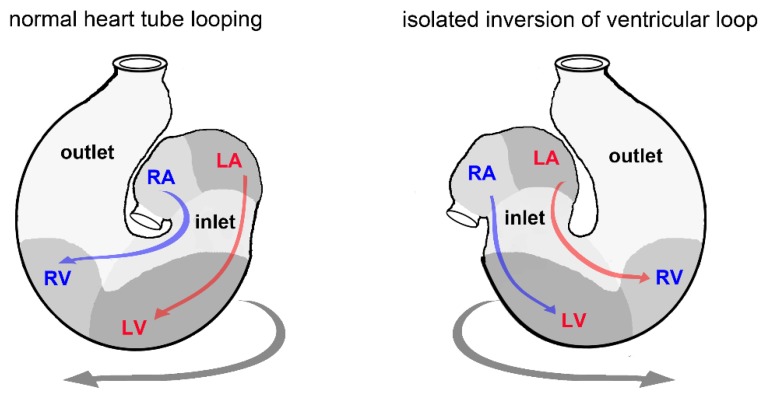
Schematic drawing illustrating the importance of cardiac looping morphogenesis for correct alignment of the intracardiac flow pathways in the four-chambered heart of lung-breathing vertebrates. The normal situation, presented on the left, shows that the normal displacement of the ventricular bend toward the right body side (d-(dextral)-looping) sets the scene for correct (concordant) alignment between the future atrial and ventricular chambers. The abnormal example, presented on the right, shows that an abnormal displacement of the ventricular bend toward the left body side (l-(levo)-looping) can set the scene for incorrect (discordant) alignment between the future atrial and ventricular chambers. LA, future left atrium; LV, future left ventricle; RA, future right atrium; RV, future right ventricle.

In view of the fact that kinking, twisting, and helical coiling of blood vessels can severely compromise the fluid dynamics of a circulatory system at macro as well as microvascular scales, the question arises why the tubular hearts of vertebrate embryos normally undergo a process of looping morphogenesis. In other words: what is the functional significance of cardiac looping? At the present time, most developmental biologists think that cardiac looping is of no significance for the pumping function of a tubular heart [52,53]. Its functional significance is generally seen at advanced stages of heart development. The prevailing view is that cardiac looping brings the building blocks of the embryonic heart tube and the developing great vessels into an approximation of their definitive topographical relationships [15,30,33,38,54,55,56]. This should set the scene for the establishment of correct connections between the future heart chambers and the great vessels during the post-looping phase of embryonic heart development (Figure 2). Kilner and co-authors proposed a further late-onset function of cardiac looping. They speculated that the curved flow pathways caused by cardiac looping might have fluidic and dynamic advantages in the mature vertebrate heart [57]. However, this idea did not receive general acceptance [58,59].

It is well known that abnormal looping of the embryonic heart can cause abnormal connections between the heart chambers and great vessels in the mature four-chambered heart of higher vertebrates [30,60]. We, therefore, cannot deny that cardiac looping plays a central role in the establishment of the definitive intracardiac flow patterns in higher vertebrates. However, does this fact justify the conclusion that the primary function of cardiac looping is to generate a topographical situation needed for the establishment of correct alignments of the future systemic and pulmonary flow pathways within the mature, multi-chambered hearts of higher vertebrates? The answer to this question may be given by comparative embryology and morphology of vertebrate hearts. In all vertebrate species studied so far, cardiac looping principally runs the same way [30,32,33,61,62,63]. This suggests that heart looping may fulfill a phylogenetically conserved function. If we now focus on the anatomy and embryology of the heart of gill-breathing fishes, we have to note that the mature heart of such fishes has only a single flow pathway and, therefore, has only two major chambers, a single atrium and a single ventricle [15,64]. Furthermore, the anlagen of these two heart chambers are correctly connected with each other as well as with the venous and arterial branches of the circulation since the beginning of heart tube formation [65]. Thus, laying down the blueprint for correct alignments of the future systemic and pulmonary flow pathways cannot be the purpose of cardiac looping in gill-breathing fishes, which represent the largest group of species among vertebrates.

What then may be the primary, phylogenetically conserved function of heart tube looping? As mentioned above, Kilner and co-authors have postulated that, compared to a linear arrangement, looped configurations of intracardiac flow pathways may have fluidic and dynamic advantages in a chambered, valve-bearing heart [57,66]. This idea of a positive, late-onset effect of cardiac looping may explain the presence of curved (s-shaped) flow pathways in the mature hearts of gill-breathing as well as lung-breathing vertebrates [15]. Unfortunately, however, this idea still awaits proof of its physical plausibility by physical experiments or numerical simulations comparing the pumping efficiency of non-looped (linear) versus looped geometries of chambered hearts [58,66]. A third late-onset benefit of cardiac looping is attributed to its torsional component which is thought to cause the “spiral” (helical) arrangement of the ventricular outflow tracts and arterial trunks normally found in the mature heart of higher vertebrates. Computational simulations of fluid dynamics and three-dimensional flow imaging have shown that, compared to a non-spiralized (linear) arrangement, the spiralized arrangement of the outflow tracts and great arteries has fluid dynamical advantages [67]. However, this function cannot explain the occurrence of heart looping in gill-breathing fishes, which do not have spiraling outflow tracts.

In our opinion, all previously published ideas about the functional significance of cardiac looping cannot give a satisfying answer to our above-mentioned question. Apart from specific faults discussed above, they all suffer from the same drawback. They exclusively postulate late-onset benefits of heart looping and, thereby, neglect the possibility that looped configurations may already change the pumping function of valveless heart tubes. Therefore, we cannot exclude the possibility that late-onset looping effects are secondary rather than primary effects. In view of the fact that blood vessel looping has the proven potential to significantly change the hemodynamics of circulatory systems (see above), we think that, if we want to uncover the primary, phylogenetically conserved function of heart looping, we should no longer focus on possible relations between form and function of the mature vertebrate heart. Instead we may focus on possible relations between form and function of the tubular embryonic heart. This would put the special case of cardiac looping into the more general context of the physiology/pathophysiology of blood vessel looping.

In the currently available literature, we found only scant and contradictory information on possible relationships between the form and pumping function of embryonic heart tubes. Based on observations on wild type zebrafish embryos, for example, Liebling and co-authors have concluded that, compared to the linear heart tube, the looped heart tube seems to be a relatively inefficient pump [68]. On the other hand, there are data from zebrafish mutants suggesting that failure of heart looping reduces the pumping efficiency of embryonic hearts [69,70].

Although most data from the adult cardiovascular system show that blood vessel looping can severely reduce the flow rate through affected vessels (see above), we personally think that we should not abandon the possibility that cardiac looping may improve the efficiency of valveless pumping in tubular hearts. This is mainly for the following reasons: (1) We can hardly believe that a biological pump of such a vital importance as the vertebrate heart should normally acquire a hemodynamically disadvantageous configuration during the initial phase of its pumping action, if the whole organism would profit from such a configuration only in the future. (2) In urochordates, which possess a valveless heart tube during their whole lifespan, the gross anatomy of the heart is characterized by bends and kinks in many species including the model organism *Ciona intestinalis* [71,72,73]. Why should such heart configurations have evolved during phylogeny if they were hemodynamically disadvantageous configurations that may reduce the fitness of an organism equipped with such a heart? (3) In all vertebrate species studied so far, the heart normally starts beating at the linear heart tube stage but hemodynamically effective blood flow usually does not begin until after cardiac looping is well-advanced [32,74,75,76,77]. This suggests that a looped rather than a straight configuration of heart tubes may support pumping. (4) The most prominent feature of vertebrate embryonic heart loops, namely their bilaterally asymmetric—”twisted”—configuration, normally is fully developed only during the embryonic period. The degree of bilateral asymmetry is normally reduced in a process called “untwisting” or “repositioning” before the developing heart reaches full morphological maturity [30,61,78,79]. In several bony fish species, this process can even completely eliminate asymmetric looping, so that the mature heart is a bilaterally symmetric structure whose curved flow pathway runs in the mid-sagittal body plane [80,81,82]. This suggests that, at least in a few species of gill-breathing fishes, some features of cardiac looping may be of functional significance only during embryonic development.

Based on the arguments outlined in the preceding paragraphs, we think that there are good reasons to postulate that the looped configurations characterizing the tubular hearts of vertebrate embryos and of some basal chordates (tunicates) represent shapes that optimize the pumping function of this type of hearts. Looping morphogenesis does not only affect the central pulsating blood vessel of vertebrate embryos. It also occurs in peripheral segments of the mature vascular system (see above), where valveless pumping phenomena are suspected to contribute to the generation of unidirectional blood flow [6,10,11,12]. We, therefore, think that the postulated beneficial effects of looping morphogenesis may work not only in valveless embryonic heart tubes but also in other periodically compressed blood vessels. If our hypothesis were correct, we would expect that embryonic heart tubes should work at a higher mechanical efficiency in a looped configuration than in a non-looped (straight) configuration. Due to the fact that the longitudinal growth of embryonic heart tubes normally is linked with their looping morphogenesis (for review see [31]), it is impossible to generate non-looped (straight) embryonic heart tubes that are exactly of the same length and diameter as their fully looped counterparts. Thus, data facilitating comparisons between the pumping performances of the two different heart tube configurations can hardly be obtained in biological models. We, therefore, decided to test the physical plausibility of our hypothesis by experimental investigations on physical pump models.

As already mentioned, the term “valveless pumping” does not define a specific physical pumping mechanism. It only characterizes pumping phenomena belonging to a group of physically diverse mechanisms, which have the common feature that they all can generate unidirectional net flow in the absence of valves. It thus makes no wonder that, in biological systems, valveless pumping phenomena cannot be attributed to only a single pumping mechanism. With respect to the valveless embryonic heart tube, for example, principally two different pumping mechanisms—“propulsive peristalsis” as well as the “Liebau effect”—are suspected to drive early embryonic blood flow since the 1950s [7,20,21,22,23,24,25,26,27,28,29]. During the past few years, two peristaltic mechanisms named “neuromechanical pumping” [25] and “biological pumping” [28] were added to the list of candidates suspected to drive early embryonic blood flow. This shows that testing of the physical plausibility of our above-mentioned hypothesis is not a simple task that may be solved by experimental investigations on only a single physical pump model. The complexity of the task is further increased by the fact that heart tube and blood vessel looping does not only generate a single form feature of potentially hemodynamic relevance, but generates a range of diverse deformations (kinking, sinusoidal bending, helical coiling, torsion). It appears that testing of our hypothesis will be a complex project in which we have to consider all biologically relevant valveless pumping mechanism as well as all hemodynamically relevant form features in a stepwise manner.

The present study is the first step of our project aimed at testing the physical plausibility of our hypothesis that looping morphogenesis may improve the efficiency of valveless pumping in periodically compressed tubular blood vessels such as the embryonic heart tube. In this study, we have compared the pumping functions of a Liebau-effect pump in a straight and a looped configuration. We have chosen to start our project with a study on a Liebau-effect pump mainly for two reasons: (1) The Liebau effect is suspected to work not only in the embryonic heart tube (see above) but also in other segments of the cardiovascular system of large animals [6,10,11,12]. Therefore, data elucidating the form-function relationships of Liebau-effect pumps may contribute not only to a better understanding of the physiology of embryonic hearts but also of the mature cardiovascular system. (2) Classical Liebau-effect pumps are technically simple devices that have only a single stationary site of active compression [7]. This makes the construction of such pumps technically easier in comparison to peristaltic pumps. The latter have multiple, non-stationary sites of active contraction moving along the whole length of the tube. Our present study, furthermore, focuses exclusively on effects of kinking and torsion. It does not consider bending or helical coiling. Kinking and torsion were chosen for the following reasons: (1) Prominent kinking occurs in the looped heart tube of vertebrate embryos [33,35,36,37] as well as in the tubular hearts of several basal chordates [71,73] and, therefore, may be regarded as a phylogenetically highly conserved form feature of valveless heart tubes. (2) Both, kinking and torsion have the experimentally proven potential to significantly change the fluid flow through affected blood vessels [48,49].

## 2. Materials and Methods

### 2.1. Liebau-Effect Pumps

Liebau-effect pumps are technically simple devices consisting of only two building elements at the minimum: (1) a valveless flexible tube of finite length, and (2) a pinching machine used for periodic compression of the flexible tube at a single, stationary site. When such a pump is placed horizontally in a basin of water, it can generate a unidirectional net flow of the fluid in its lumen if it is periodically compressed at an asymmetric position along its length [8,83,84]. Periodic compressions at the point of symmetry (middle of the tube) do not generate a unidirectional net flow. The same flow phenomena are observed if the pump is connected to a fluid-filled pipe system [9,11]. Analyzing the behavior of a Liebau-effect pump in a closed pipe system has shown that such a pump can generate unidirectional fluid flow only if there is a mismatch of impedance present at its two ends with which it is connected to the pipe system [11]. Consequently, this flow is named impedance defined flow [11] and Liebau-effect pumps are frequently named impedance pumps [85,86,87,88]. Currently, the pumping effect generated by a Liebau-effect pump is explained by wave dynamics of its flexible wall [85,89,90]. Periodic compressions of its wall at a single, stationary site generate bidirectional pairs of passive mechanical waves that travel along its wall from the point of compression towards its two ends, where they are partially reflected due to the mismatch of impedance. In cases of asymmetrically positioned contraction sites, the sum interaction of emitted and reflected waves is said to cause unidirectional net flow of the fluid contained in the pump. The net flow generated by a Liebau-effect pump has several unique characteristics that are well explained by wave dynamics [85,89,90]. There is, for example, a non-linear relationship between the compression frequency and flow rate. Furthermore, the direction of net flow not only depends on the position of the compression site with respect to the ends of the pump, but also, additionally, depends on the compression frequency. Thus, modulating the compression frequency can induce flow reversals.

### 2.2. General Design of the Experimental Apparatus

**Figure 3:**
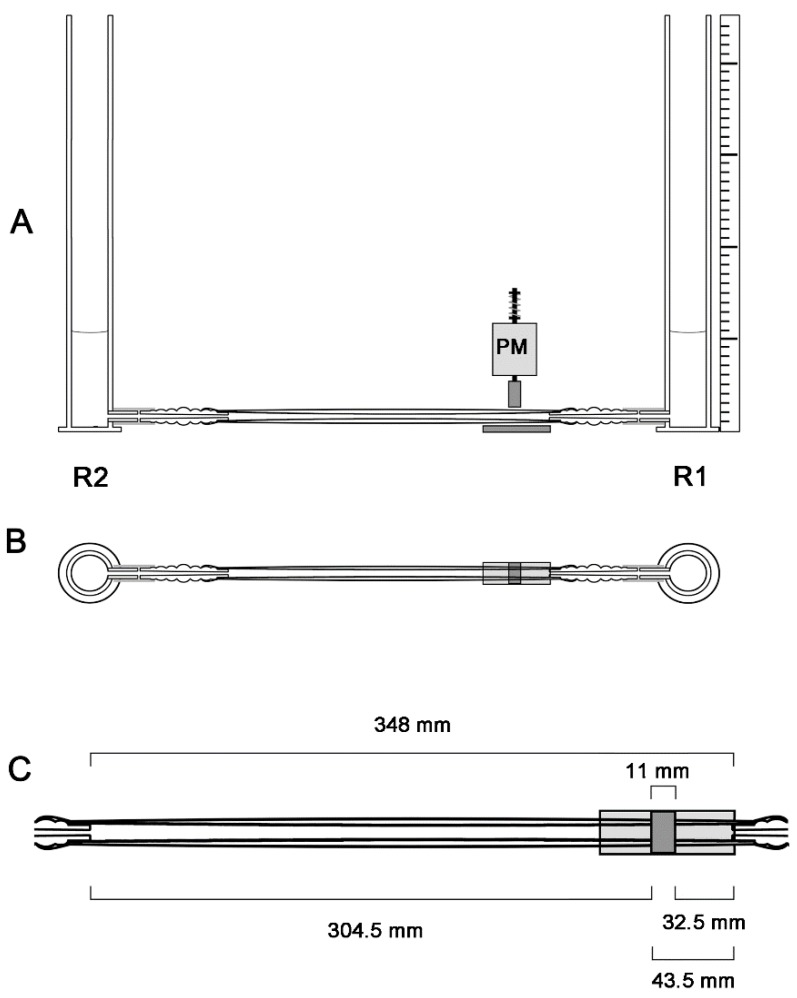
Graphical presentation of the design and dimensions of our experimental apparatus in the straight tube configuration. (**A**) Pumping system in a frontal view; (**B**) Pumping system from the upside; (**C**) Pump (upside view) in a higher magnification. PM, pinching machine; R1, reservoir 1 (upstream reservoir); R2, reservoir 2 (downstream reservoir).

**Figure 4:**
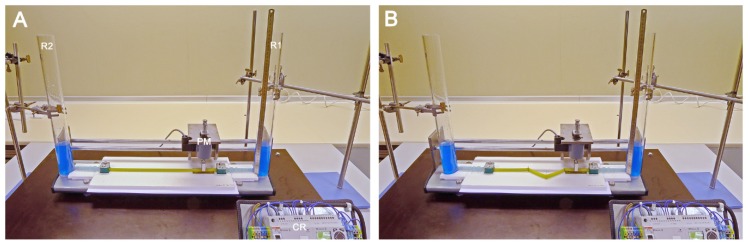
Photographs of our experimental apparatus in the straight tube (**A**) and looped tube configuration (**B**). For better visualization, working fluid was stained with blue color. CR, control relay; PM, pinching machine; R1, reservoir 1 (upstream reservoir); R2, reservoir 2 (downstream reservoir).

**Figure 5:**
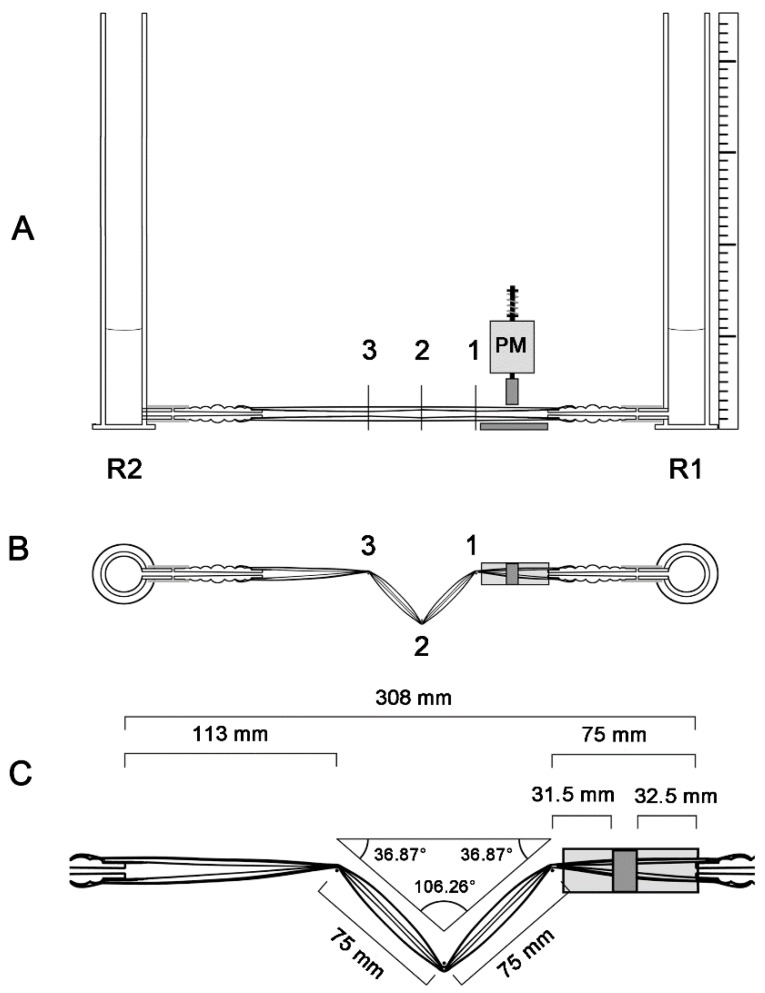
Graphical presentation of the design and dimensions of our experimental apparatus in the looped tube configuration. The looped tube configuration is characterized by the presence of three kinks (positions marked by labels 1, 2, and 3) and two torsions (positions marked by labels 1 and 3). (**A**) Pumping system in a frontal view; (**B**) Pumping system from the upside; (**C**) Pump (upside view) in a higher magnification. PM, pinching machine; R1, reservoir 1 (upstream reservoir); R2, reservoir 2 (downstream reservoir).

In the present study, we have built an experimental apparatus that was used for analyzing the pumping functions of a Liebau-effect pump in two different geometric configurations: (1) in a straight tube configuration (Figure 3 and Figure 4A); and (2) in a “looped” tube configuration, which was characterized by the presence of three kinks (Figure 4B and Figure 5). In previous experimental studies, the behavior of Liebau-effect pumps was analyzed either in closed pipe systems [9,11,91,92,93,94] or in open pipe systems [5,8,85,86,87,88,92,95,96]. The experimental apparatus used in the present study was an open pipe system comprising two reservoirs (acrylic glass cylinders) and one Liebau-effect pump in the middle (Figure 3, Figure 4 and Figure 5). An open pipe system was chosen since changing the geometric configuration of a tubular pump of fixed length from a straight to a looped configuration is accompanied by a reduction of the linear distance between its two ends. Thus, in a closed pipe system, looping of a tubular pump can be done only in combination with changes in the dimensions of the rest of the system (lengthening of rigid pipe or decreasing the radius of rigid pipe curvatures), which may have unwanted influence on the pumping efficiency. An open two-reservoirs system can be adapted to pump looping simply by reducing the linear distance between the two reservoirs, which does not alter the dimensions of the rigid components of the system.

The pump used in our experiments was a multilayered flexible tube. Its design was inspired by the multilayered structure of the embryonic heart tube. Previous studies have shown that the multilayered structure of the embryonic heart tube supports the pumping action of peristaltic as well as Liebau-effect pumps [97,98]. The beneficial effect of a multilayered pump design was also confirmed in own tests, which were carried out before starting our experimental series (data not shown). The multilayered pump used in our experiments was made of an outer silicone rubber tube (“Penrose surgical drain” size 3, wall thickness 0.13 mm, inner inflated diameter 10 mm, length ~ 410 mm, Rüsch GmbH, Kernen, Germany), representing the myocardial wall of the embryonic heart tube, and a smaller inner silicone rubber tube (“Penrose surgical drain” size 1, wall thickness 0.13 mm, inner inflated diameter 6.33 mm, length ~ 410 mm, Rüsch GmbH), representing the endocardial wall of the embryonic heart tube. The space between the outer and inner tubes was filled with distilled water (8.56 mL at 20 °C), representing the cardiac jelly layer of the embryonic heart tube. The ends of the pump were connected to rigid tubing connectors made of polypropylene (neoLab-Universal Tubing Connector for tubing with 5–17 mm inner diameter). At the connecting sides, the inner and outer rubber tubes overlapped the ends of the tubing connectors for ~22 mm (inner rubber tube) and ~31 mm (outer rubber tube) and were secured with rubber rings to prevent leakage. Thus, the length of the flexible portion of the Liebau-effect pump (distance between the points of fixation of the outer elastic tube to the rigid tubing connectors) was 348 mm in the straight configuration (410 mm − 2 × 31 mm). Tubing connectors were used to connect the pump to two reservoirs at each end of the experimental apparatus.

The two reservoirs were made of vertically held acrylic glass cylinders (457 mm height). They had an outer diameter of 50 mm and an inner diameter of 40 mm, and a wall thickness of 5 mm. Each reservoir had a horizontally held inlet/outlet tube at its base (length 16 mm, outer diameter 9 mm, inner diameter 5 mm). The position of the opening of the inlet/outlet tube was 11 mm above the bottom of the reservoir. The inlet/outlet tubes were connected to the tubing connectors of the pump via silicone tubes (inner diameter 6 mm, wall thickness 1 mm, length 35 mm, overlap ~25 mm (tubing connector) and ~10 mm (inlet/outlet tube)).

The system was filled with working fluid (20 °C) to the level of 110 mm in the two reservoirs. For analyzing the pumping function under conditions of macrovascular flow, the system was filled with distilled water. For analyzing the pumping function under conditions of microvascular flow (“viscous flow” or “low Reynolds number flow”), which are the conditions present in the embryonic circulation during cardiac looping [23,26,99,100,101], the system was filled with a viscous fluid (mixture of 50% water and 50% Karo brand light corn syrup). The rational for using Newtonian fluids instead of non-Newtonian fluids (e.g., blood) is the fact that embryonic blood behaves as a Newtonian fluid [102,103]. Increasing the viscosity of the working fluid of a pipe system driven by an oscillating pump system can be used to simulate the flow conditions within small blood vessels in models of macrovascular scales [29,101,104]. This downscaling approach reduces the values of two dimensionless numbers in fluid mechanics, which are used to compare the flow behavior at different size scales (e.g., macro and microcirculation). These are the Reynolds number and Womersley number. Both numbers are less than 1 in the microcirculation (see below).

### 2.3. Pinching Machine and Modes of Periodic Compression

For periodic compression of our Liebau-effect pump, we built a pinching machine driven by an electromagnetic push-pull type actuator (Asco 24V DC, Joucomatic GmbH, Ölbronn-Dürrn, Germany). The actuator was supplied and controlled by a device comprising an AC-DC converter and a control relay (Moeller easy 400-POW, Moeller easy 512-DC-R, Eaton Moeller GmbH, Bonn, Germany). The pinching machine was positioned vertically above the horizontally held pump, which, in its turn, rested on a hard horizontal plate (Figure 3 and Figure 5). During a short rectangular current impulse of 0.1 s, the electromagnetic actuator produced a linear mechanical force that pushed a cuboid–shaped steel block (11 mm width, 27.5 height, 24 mm depth) down onto the pump. Thereby, the pump was compressed against the hard horizontal plate. Upon the end of the electric current, the pinching block was pulled back upward by elastic recoiling of a steel spring, which was a component of the actuator and had been compressed during the preceding action of the electromagnet. Corresponding to the electric impulse driving the mechanical action of the electromagnetic actuator, the compression wave had a rectangular shape and a length of ~0.1 s. At its lowest point, the pinching block was 1 mm above the horizontal plate and closed off the lumen of the pump completely. At its highest point, the pinching block was 9.5 mm above the horizontal plate. Here, the pinching block did not contact the fully inflated pump under the present experimental conditions. The width of the compression zone of the pump was 11 mm, corresponding to the width of the pinching block. For the generation of unidirectional net flow, the compression zone was placed at an asymmetric position along the length of the pump. In all experiments carried out in the present study, the compression zone was at the same place, which extended from 32.5 mm to 43.5 mm away from either end of the pump (opening of the rigid tube connectors). Tests that were carried out in preparation of our experimental series had shown that positioning of the compression zone at this site provides optimal pumping performance in the straight tube configuration (data not shown).

Experiments were carried out for compression frequencies of 0.5, 1.0, 1.5, 2.0, 2.5, and 3 Hz. This range of compression frequencies was chosen since it covers the physiological range of heart rates found in vertebrate embryos during the phase of cardiac looping (see Figure 292 in [105]; Figure 2 in [14]; Figure 1 in [106]; Figure 1 in [107]; Table 1 in [23]; Figure 7 in [108]). Compression waves had a rectangular shape and a length of ~0.1 s (see above).

### 2.4. Geometric Configurations of the Liebau-Effect Pump

The main purpose of our study was to test the physical plausibility of our hypothesis that looping morphogenesis may improve the efficiency of valveless pumping in tubular blood vessels such as the vertebrate embryonic heart tube. We have, therefore, analyzed the pumping functions of our Liebau-effect pump in two different geometric configurations: (1) in a straight tube configuration; and (2) in a looped tube configuration.

Design and dimensions of the experimental apparatus in the straight tube configuration of our Liebau-effect pump are already described above and are summarized in Figure 3 and Figure 4A.

Design and dimensions of the experimental apparatus in the looped tube configuration of our Liebau-effect pump are shown in Figure 4B and Figure 5. In the looped tube configuration, only the spatial course of the pump was changed in conjunction with shortening of the linear distance between the two reservoirs, while all other structural parameters (e.g., position of compression zone, length of pump) were the same as in the straight configuration. The looped configuration was characterized by the presence of three kinks within the course of the pump. The three-kink configuration was inspired by: (1) the V-shaped form of the valveless heart tube of basal chordates, such as *Ciona intestinalis*; (2) the zigzag course of kinked arterial or venous segments in human cardiovascular systems; and (3) the C-shaped form of valveless heart tubes of vertebrate embryos. The position of the three kinks was physically defined by three metal pins, which were vertically inserted into the horizontal plate under the experimental apparatus. These pins acted as deviation points for the course of the pump. Their positions are shown in Figure 5B,C. Since the pump was built from collapsible tubes, which have a flat shape at compression, the deflection of the horizontally held pump around vertically oriented pins did not only produce kinking but additional produced torsions of the flow path of the pump. Kinking led to a subdivision of the pump into four segments. The first three segments were of the same length (~75 mm), while the fourth segment was slightly longer than the others (~113 mm). The compression zone was in the middle of the first segment, while the second and third segment formed the limbs of the V-shaped portion of the pump. Tests that were carried out in preparation of our experimental series had shown that positioning of the kinks at the three above-mentioned sites provide optimal pumping performance in the looped tube configuration (data not shown).

### 2.5. Measurements and Calculations

During the pumping experiments, the changing fluid level (∆h) in one of the two reservoirs was measured continuously using a video camera (Blaupunkt, Hildesheim, Germany) interfaced to a PC. Experiments were stopped when the measured fluid level had reached a steady state (∆hmax). At this point, the maximum pressure head (∆pmax) is reached so that no further net flow is generated by the pump and the fluid level oscillates with the pumping frequency. Based on the video data, the maximum pressure head (expressed in mm H_2_O) reached in each experiment was calculated as follows: (1) For experiments using distilled water as working fluid, ∆pmax [mm H_2_O] = 2 × ∆hmax [mm]. (2) For experiments using a 1:1 mixture of distilled water and light corn syrup (density 1.233) as working fluid, ∆pmax [mm H_2_O] = (2 × ∆hmax [mm]) × 1.233.

**Figure 6:**
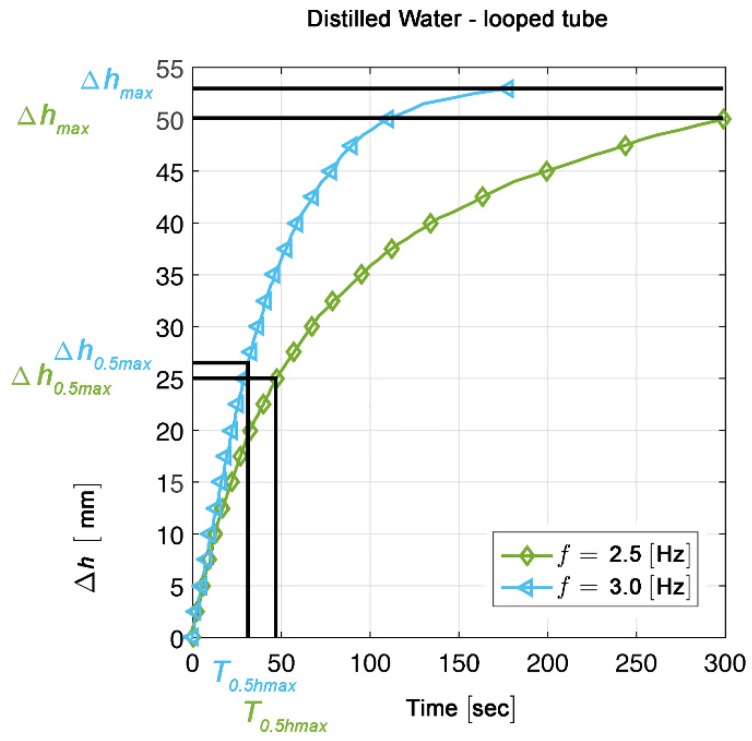
Diagram illustrating the method used for definition of the phase of approximately linear rise of the fluid level in the downstream reservoir. Changes in the fluid levels of one reservoir (∆h/∆t) are shown for two experiments (distilled water, looped tube, compression frequencies 2.5 Hz and 3.0 Hz). The phase of approximately linear rise of the fluid level in the downstream reservoir was defined as lying between the starting point of the experiment (T0) and the time point when half of the maximum fluid level was reached in the downstream reservoir (T0.5hmax).

For estimation of average flow rates (FR) and average flow velocities (U), the changes in the fluid level in one reservoir were continuously recorded over time (∆h/∆t). FR and U were calculated for the phase of approximately linear rise of the fluid level in the downstream reservoir (Figure 6). This phase was defined as lying between the starting point of the experiment (T0) and the time point when half of the maximum fluid level was reached in the downstream reservoir (T0.5hmax). The fluid volume displaced during this time (∆vol0.5max) was calculated as follows: ∆vol0.5max [mm^3^] = (0.5 × ∆hmax [mm]) × (π × r2 [mm]), where r is the inner radius of the reservoirs (= 20 mm). The average flow rate was estimated as FR = ∆vol0.5max [mm3]/T0.5hmax [s]. The average flow velocity in the reservoirs (r = 20 mm) was calculated as follows: UR = ∆h0.5max [mm]/T0.5hmax [s], and the average flow velocity in the pump (r = 3.1665 mm) was calculated as follows: UP = (∆h0.5max [mm]/T0.5hmax [s]) × 39.89.

The kinematic viscosity of the working fluids was measured at 20 °C using an Ubbelohde viscometer. The kinematic viscosity (kv) of distilled water was 1.004 mm^2^/s, and the kinematic viscosity (kv) of the water-syrup mixture was 10.7625 mm^2^/s.

The flow behavior of fluids within biological pipes depends on several dynamic flow parameters, such as flow velocity or pulsatile flow frequency. The fluid mechanical effects of these parameters change with the size of the pipe system in non-linear fashions. This means, for example, that the same flow speed or pulsation frequency will cause different effects in pipes, which are of the same shape but of different size. As a consequence, the results from experimental models of pipe systems, such as our Liebau-effect pumps, can hardly be correlated with the real situation unless scaling factors are taken into consideration. In fluid mechanics, correlations between the flow behaviors in pipes of different size (e.g., experimental model vs. reality) are usually made on the basis of dimensionless numbers, which are regarded as qualitative indicators for the flow behavior. Such indicators are the Reynolds number and the Womersley number.

The Reynolds number is an expression of the ratio of inertial forces to the viscous forces within a fluid moving along a surface (e.g., wall of a pipe system). It indicates when turbulent flow will occur in particular situations. When the Reynolds number is large, as is common in the case of macrocirculation, the flow is dominated by inertial forces, which tend to produce turbulent flow. When the Reynolds number is small (≤1), as is common in the case of microcirculation, the flow is dominated by viscous forces, which tend to produce laminar flow. Based on the above-mentioned calculations and measurements, the Reynolds numbers of the flows within the inner tube of the pump (inflated diameter 6.333 mm) were calculated as follows: Re = U × Dh/kv, where U is the average flow velocity (see above), Dh is the hydraulic diameter, and kv is the kinematic viscosity of the working fluid (see above).

The Womersley number is used as a qualitative indicator for the flow behavior in biological pipes with unsteady (pulsatile) flow, e.g., arteries or periodically compressed blood vessels. It is an expression of the ratio of the unsteady forces (pulsatile flow frequency) to the viscous forces. When the Womersley number is large (≥10), the fluid flow is dominated by unsteady (oscillatory) inertial forces and the velocity profile is flat. When the Womersley number is low (≤1), as is common in the case of microcirculation, viscous forces dominate, velocity profiles are parabolic and the centerline velocity oscillates in phase with the driving pressure gradient. Based on the above-mentioned calculations and measurements, the Womersley numbers were calculated as follows: Wo = Dh √ (ω/kv), where ω is the frequency, Dh is the inner pump diameter, and kv is the kinematic viscosity of the working fluid (see above).

## 3. Results and Discussion

The present study was conducted to test the physical plausibility of our hypothesis that looping morphogenesis may improve the efficiency of valveless pumping in vertebrate embryonic heart tubes as well as in other periodically compressed tubular blood vessels. We have analyzed the pumping performances of a Liebau-effect pump in two different geometrical configurations, (1) a straight tube configuration; and (2) a looped tube configuration, which was characterized by the presence of three kinks. The Liebau effect is a valveless pumping phenomenon that is suspected to contribute to the generation of unidirectional blood flow in large blood vessels such as the aorta [12], as well as in small blood vessels such as the vertebrate embryonic heart tube [6,8,20]. It is well known that the characters of blood flow can differ considerably between large and small blood vessels. The former is largely determined by inertial forces, as indicated by relatively high Reynolds and Womersley numbers, while the latter is largely determined by viscous forces, as indicated by relatively low Reynolds and Womersley numbers [109]. The analyzes of the pumping performances of our Liebau-effect pumps, therefore, were carried out in two different sets of physical experiments; one which was intended to simulate the flow conditions in large blood vessels, and another one which was intended to simulate the flow conditions in small blood vessels. The dimensions of the experimental apparatus used in the present study correspond to those of relatively large blood vessels, so that the usage of distilled water as working fluid was regarded as sufficient for simulating the character of blood flow at macrovascular scales (experimental set 1). Microvascular flow is largely determined by viscous forces. Its character can be approximated in a macrovascular pump system by increasing the viscosity of the working fluid [101,104]. This approach does not only reduce the Reynolds number but also the Womersley number [29,101,104]. Our analyzes of the behavior of Liebau-effect pumps at the scales of small blood vessels, therefore, could be carried out in the same experimental apparatus simply by using a 1:1 mixture of distilled water and corn syrup as working fluid (experimental set 2). Since our study was intended to provide data that should be close to the physiological conditions of the circulatory systems of vertebrates, we did not test our Liebau-effect pumps over a wide range of compression frequencies, as has been done in many previous studies on this kind of pumps, but confined the range of tested compression frequencies to the range of heart rates normally found in vertebrate embryonic hearts during the phase of looping morphogenesis (0.5–3 Hz; [14,105,106,107,108]). This range of compression frequencies includes the physiological range of heart rates in the postnatal human heart.

As expected, our experimental data disclosed some remarkable differences between the performances of a Liebau-effect pump in a straight and a looped configuration. There were, however, also a few characteristics in the pumping behavior that did not differ between the various experimental sets. Since these characteristics are important for evaluating the possible functional relevance of the Liebau effect in the embryonic and postembryonic cardiovascular system of human beings, we would like to present and discuss these characteristics before we will come to the differences.

### 3.1. Minimum Compression Rate Needed for Generation of Unidirectional Net Flow

**Figure 7:**
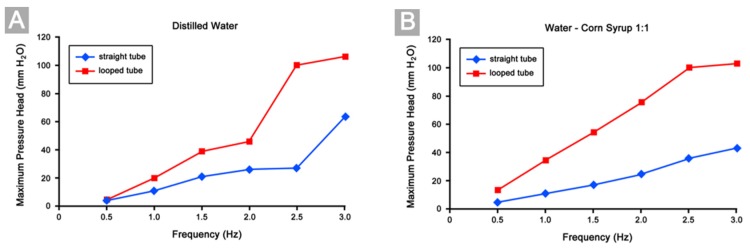
Diagrams showing the maximum pressure heads (∆pmax) reached in our experiments. (**A**) Results of low viscous fluid (distilled water) pumping; (**B**) Results of high viscous fluid (1:1 mixture corn syrup/distilled water) pumping.

**Figure 8:**
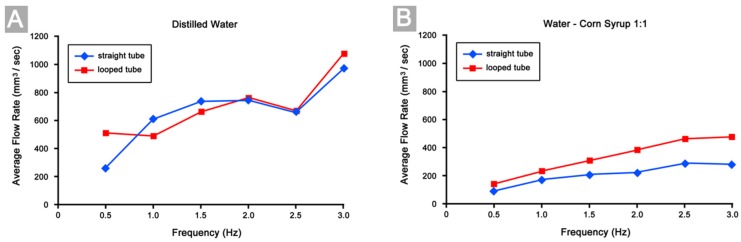
Diagrams showing the average flow rates (FR) reached in our experiments. (**A**) Results of low viscous fluid (distilled water) pumping; (**B**) Results of high viscous fluid (1:1 mixture corn syrup/distilled water) pumping.

In a relatively large number of previous experimental studies, the performances of Liebau-effect pumps were tested over a range of compression frequencies that exceeded the range of heart rates normally found in the embryonic and postembryonic human heart. The graphical presentations of the data obtained in these studies show that Liebau-effect pumps seem to generate high net flow rates only at compression frequencies near to 5 Hz, while compression frequencies below 3 Hz seem to produce only very small flow rates [85,87,89,93,94]. Moreover, in one study, it was found that Liebau-effect pumps did not produce any net flow at compression frequencies lower than 8 Hz [86]. This may suggest that the Liebau effect cannot generate effective unidirectional fluid flow at compression frequencies corresponding to the normal heart rates of embryonic and postembryonic human beings. However, in all our present experiments unidirectional net flow was produced at all compression frequencies tested (Figure 7 and Figure 8).

Our present observations, therefore, show that a Liebau-effect pump can generate unidirectional net flow at compression frequencies corresponding to the heart rates normally found in the embryonic and postembryonic human heart. This corresponds to observations made by several other experimenters [9,11,91,95,110]. At first sight, the failure to generate significant net flow at heart-specific compression rates, observed in some of the above-mentioned studies on Liebau-effect pumps, may appear as an enigma. We have to note, however, that the fluid mechanical effects of unsteady forces, such as oscillating compressions, depend on the size of the pump system. This means that the compression frequencies applied to systems of different sizes cannot be directly compared with each other. The frequencies need to be transformed into non-dimensional frequency parameters, such as the Womersley number, before such comparison can be made. Therefore, the above-mentioned differences in the performances of Liebau-effect pumps may be explained simply by differences in the scaling of the pump systems. Comparison of the Womersley numbers would be the correct way to uncover true differences or similarities in frequency-flow relations. Unfortunately, Womersley numbers were not used in the majority of previously published studies on Liebau-effect pumps. This hampers quick and easy comparisons of the results of these studies. To illustrate the value of usage of the Womersley number, we should note that the above-mentioned failure to generate significant net flow at the lower end of frequency-flow curves of Liebau-effect pumps may be explained by the dominance of viscous forces, which is indicated by small Womersley numbers. Baird and co-workers [24] have reported that the passive wave dynamic driving the Liebau-effect is overly damped if viscous effects are large relative to the stiffness of the wall of the pump system. For the wall stiffness considered in their model, they found that little to no net flow was generated for Womersley numbers ≈ 1. The Womersley numbers encountered in our present experiments ranged from 1.36 to 10.95 (Table 1) and, therefore, exceeded the “critical” number reported by Baird et al. [24]. Although we do not know the wall stiffness of our pump system, we think that this fact might explain that we did not observe any failure in generating significant net flow within the range of tested compression frequencies.

### 3.2. Relation between Compression Frequencies and Stability of Flow Direction

Previous experimental studies on Liebau-effect pumps have shown that modulating the compression frequency can induce flow reversals in a seemingly unpredictable manner [85,89,90], and flow reversals were observed even within a range of compression frequencies corresponding to the heart rates normally found in vertebrate embryos during the phase of cardiac looping [95]. It has, therefore, been questioned whether the Liebau effect may be a good candidate to explain unidirectional blood flow in the valveless heart tube of vertebrate embryos [7]. In all our present experiments, however, we did not observe any reversal of the direction of net flow within the range of compression frequencies tested (Figure 7 and Figure 8). This shows that Liebau-effect pumps can generate a robust unidirectional fluid flow within the range of the steadily increasing heart rates normally found in vertebrate embryos during the phase of cardiac looping. At the present time, we do not know the factors responsible for stabilization or destabilization of the flow direction within the range of compression frequencies tested in our experiments. Based on numerical analyzes, Koslovsky and co-workers have found that high viscous flow is a stabilizing factor [27]. This observation may explain the robustness of unidirectional flow found in our second set of experiments, where we used a 1:1 mixture of water and corn syrup as working fluid. It can hardly explain, however, the absence of flow reversals in the first series of our experiments, where we used distilled water as working fluid.

### 3.3. Relation between Flow Direction and Position of the Compression Zone

**Figure 9:**
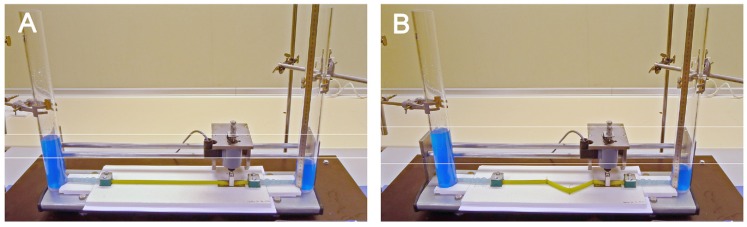
Photographs illustrating the direction of net flow generated in our pump systems and the increase in maximum pressure head (∆pmax) reached by the looped tube (**B**) as compared to the straight tube (**A**). For better visualization, working fluid was stained with blue color.

Previous studies on Liebau-effect pumps have consistently shown that the generation of unidirectional net flow depends on an asymmetric positioning of the compression zone along the length of the pump. With respect to the relation between the positioning of the compression zone and the direction of flow, however, data obtained at corresponding ranges of compression frequencies provide a divergent picture. On the one hand, net flow was found to run from the end remote from the compression zone toward the end close to the compression zone [2,5,6,8,9,10,11,93,110]; while on the other hand, net flow was found to run exactly in the opposite direction [85,89,91,111]. This suggests that the direction of net flow generated by a Liebau-effect pump not only depends on the compression frequency and the position of the compression zone but also, additionally, depends on further, hitherto poorly defined factors. In our present experiments, the direction of the unidirectional net flow was always from the end close to the compression zone toward the end remote from the compression zone, so that we could say that the compression zone was located close to the inflow of our Liebau-effect pump (Figure 9). Such a relationship between the flow direction and the position of the compression zone is of interest since it may correspond to the situation in vertebrate embryonic heart tubes. Here several researchers have reported that the metabolic and contractile activities of looped embryonic heart tubes are not evenly distributed along their length. Such heart tubes rather seem to possess a single—”kick start”—center, which is positioned close to its inflow end and shows a higher metabolic activity and faster contraction than the rest of the heart [20,38,112,113].

### 3.4. Differences in Pumping Performance

The above-mentioned characteristics in the behavior of our Liebau-effect pump obviously were not affected by a change of its geometrical configuration from a straight to a looped tube. However, with respect to the pumping performance, as expressed by the maximum pressure heads and the average flow rates generated by a pump, our experiments disclosed striking differences between the straight tube and looped tube configurations of our Liebau-effect pump. Compared to the straight tube configuration, the looped tube configuration was able to generate higher maximum pressure heads (Figure 7) and higher average flow rates when tested at the same compression frequencies (Figure 8). These two effects were especially significant in cases of high viscous fluid pumping (experimental set 2). Here, both, the maximum pressure heads and the average flow rates generated by the looped tube configuration were always significantly higher than the corresponding values generated by the straight tube configuration (Figure 7B and Figure 8B). Increase in the maximum pressure heads achieved by the looped configuration ranged from 137% to 214%, while increase in the average flow rates ranged from 31% to 72%. In cases of low viscous fluid pumping (experimental set 1), however, the picture was not so simple. Here, only the values of the maximum pressure heads generated by the looped tube configuration always exceeded those generated by the straight tube configuration (Figure 7A; increase ranged from 25% to 270%). The average flow rates, on the other hand, did not show a consistent pattern. The flow rates generated by the looped tube configuration sometimes were similar (at 2.0 Hz (+1%) and 2.5 Hz (+2%)), higher (at 0.5 Hz (+96%) and 3 Hz (+10%)), or even lower (at 1.0 Hz (−20%) and 1.5 Hz (−10%)) than those generated by the straight tube configuration (Figure 8A).

These results show that, for the parameters tested in the present study, kinking and torsion of an initially straight Liebau-effect pump can lead to a significant improvement of its pumping efficiency. It should be emphasized, that this effect was especially significant in cases of high viscous fluid pumping (experimental set 2). This is of special interest because dominance of viscous effects over the stiffness of the pump wall is said to damp the passive wave dynamics driving the Liebau effect [24] with the final consequence that traditional (straight) Liebau-effect pumps do not work very efficient at low Reynolds and Womersley numbers [24,93,114,115]. Our finding that looping morphogenesis can improve the efficiency of Liebau-effect pumps for pumping of low as well as high viscous fluids suggests that kinking and torsion may counteract the damping effects of viscous forces and, thereby, may make Liebau-effect pumps suitable for pumping of high viscous fluids. The passive wave dynamics driving the Liebau effect depend on the relation between viscous forces and wall stiffness [24]. It is, therefore, tempting to speculate that, in cases of dominance of viscous forces, kinking and torsion may counteract the damping of passive wave dynamics by increasing the stiffness of the pump wall. Future studies may test this speculation. We should note, however, that the Reynolds and Womersley numbers encountered in our present experiments of high viscous fluid pumping were higher than 1 (Table 1 and Table 2). Dominance of viscous forces is commonly reached when the Reynolds and Womersley numbers are ≤1. We, therefore, cannot exclude the possibility that the beneficial effects of kinking are limited to situations where the Reynolds and Womersley numbers are larger than 1.

### 3.5. Mechanisms Responsible for Improvement of Pumping Efficiency

Having demonstrated the beneficial effects of kinking and torsion on the pumping performance of a Liebau-effect pump, two questions arise: (1) how do these deformations lead to an improvement of the pumping efficiency? (2) May our findings have any relevance for the understanding of fluid transport in biological conduits and, thereby, may provide evidence supporting our hypothesis that looping morphogenesis may improve the efficiency of valveless pumping in the cardiovascular system of vertebrates? With respect to the first question, we have already speculated that the improvement of the pumping efficiency may result from changes of the relation between viscous forces and wall stiffness (see previous paragraph). Besides this idea, we can offer three further ideas, which are based on three form features characterizing the looped configuration of our Liebau-effect pump. These were (a) temporary complete occlusions of the lumen of the tubular pump at the sites of kinking, which were only opened when traveling waves, evolved during the pumping action, passed the sites of kinking; (b) asymmetric positioning of the kinks along the length the pump; and (c) a subdivision of the pump into four, serially aligned segments. With respect to the first form feature, it is tempting to speculate that the kinked portions of the tube may have acted in a valve-like or sphincter-like manner. This hypothesis is not a really new idea. A valve-like function was previously attributed to kinked segments of blood vessels by several researchers. Brocas and co-workers [116] have speculated that the kink at the apex of the V-shaped heart tube of the tunicate *Ciona intestinalis* might function as a valve. Radiologists explained failure of filling of cerebral arteries with contrast medium by a valve mechanism due to kinking of the internal carotid artery [117], and neurosurgeons have speculated that the zigzag course of the valveless radicular veins of the spinal cord act in a valve-like manner [118]. The valve function hypothesis is also attractive since Moser and co-workers [11] have shown that the addition of a valve to a fluid conducting system driven by a Liebau-effect pump can significantly improve its pumping efficiency. With respect to a possible role of the second form feature,—asymmetric positioning of the kinks—, we should note that it is a well-known fact that the Liebau-effect depends on asymmetric positioning of the active compression site along the length of the pump [8,83,84]. Thus, asymmetric positioning of kinks does not introduce a new kind of morphological quality to our Liebau effect pump but merely has added a further asymmetry to the pump and thereby has increased the degree of asymmetry along its length. It is tempting to speculate that asymmetric positioning of the kinks may reinforce the wave dynamics responsible for the generation of unidirectional net flow. With respect to the third form feature—pump segmentation—, one might speculate that a Liebau-effect pump of such a design does not function as a single Liebau-effect pump but rather act as a chain of multiple, serially aligned Liebau-effect pumps. This speculation was inspired by experimental investigations of Lee and co-workers [87,88,119], who showed that a multi-stage Liebau-effect pump could generate higher pressure heads and higher flow rates than a single-stage Liebau-effect pump. Future studies may clarify how kinking improves the pumping efficiency of Liebau-effect pumps and whether one of the four above-mentioned mechanism may contribute to this effect.

### 3.6. Biological Relevance

With respect to the question of a possible biological relevance of our data, we should note that answering of this question is intimately related to answering of the more general question whether valveless pumping phenomena, such as the Liebau effect or propulsive peristalsis, may contribute to the propulsion of body fluids. With respect to the mature cardiovascular and lymphatic systems of vertebrates, unidirectional fluid flow is traditionally attributed to the action of valve-supported pumps, only [120,121,122,123]. Such pumps have been categorized as central (the central heart) or peripheral pumps (blood vessels such as valve-bearing veins [120]). Only a few researchers have pointed to the possibility that valveless pumping phenomena may also contribute to the generation of unidirectional blood flow under physiological as well as patho-physiological conditions [10,11,12]. Unfortunately, this idea did not find general acceptance. The historical neglect of the possible action of valveless pumping phenomena in mature cardiovascular systems may be explained, in part, by the previous lack of knowledge about form features supporting the function of valveless pumps. Our present data suggest that kinking or a zigzag course of blood vessels may be form features characterizing vessels acting as highly efficient Liebau-effect pumps. Thus, search for these form features may help to identify those portions of the vascular tree, which may function as valveless pumps. In the past, the presence of macro as well as microvascular kinking was exclusively suspected to exert deleterious effects on organ and tissue perfusion [41,44,45,50,51]. Our present data may lead to a rethinking about the functional significance of blood vessel kinking.

The possible contribution of valveless pumping phenomena to the hemodynamics of the mature cardiovascular system of human beings obviously is an unresolved matter. It is a generally accepted view, however, that, during early embryonic development, blood flow is exclusively driven by a valveless pumping mechanism. The same holds true for the tubular hearts of basal chordates (tunicates), which frequently show prominent kinking (e.g., *Ciona intestinalis*). In view of our present data, it is, therefore, tempting to interpret heart tube kinking and torsion as form features optimizing its pumping efficiency. We have to note, however, that our data only show that these form features can improve the efficiency of Liebau-effect pumps. At the present time, it is not clear whether valveless heart tubes work as Liebau-effect pumps or as peristaltic pumps (see Introduction). In a seminal paper, Forouhar and co-workers [20] have stated that these two types of valveless pumps differ considerably from each other with respect to their pumping behavior. The generation of pulsatile flow, of a peak flow velocity that can exceed the speed of the elastic waves traveling along the tube wall, and a non-linear relationship between compression frequency and flow rate were regarded as distinctive characteristics of Liebau-effect pumps. The behavior of peristaltic pumps, on the other hand, was thought to be characterized by the generation of continuous flow, a peak flow velocity that did not exceed the speed of the propagating contraction wave, and a linear relationship between compression frequency and flow rate. Forouhar and co-workers [20], therefore, have suggested that the specific behavior of a Liebau-effect pump might be used to identify such pumps in living organisms. They analyzed the pump action of the valveless heart tube of zebrafish embryos and reported that it was characterized by pulsatile flow, peak flow velocities that could exceeded the speed of the traveling waves, and a non-linear relationship between heart rate and flow rate. It was, therefore, concluded that embryonic heart tubes might work as Liebau-effect pumps rather than peristaltic pumps. During the past few years, however, several studies have shown that the above-mentioned characteristics of Liebau-effect pumps can be generated not only by this kind of pumps but also by peristaltic pumps [21,29]. It appears that the two different valveless pumping phenomena seem to share more functional characteristics than previously thought. The initially quoted “characteristics” of Liebau-effect pumps obviously cannot be used as distinguishing criteria between Liebau effect and peristaltic-driven flow. There seems to exist, however, at least one functionally important difference between peristaltic and Liebau-effect pumps, which might be used to answer the question whether embryonic heart tubes work as Liebau-effect or peristaltic pumps. This is the potency to generate significant net flow under the conditions of dominance of viscous forces [24]. Vertebrate embryonic heart tubes are small blood vessels that work at very low Reynolds and Womersley numbers [23,24,99,102]. The Reynolds and Womersley numbers calculated for such hearts commonly are less than 1. This means that the pumping mechanism driving early embryonic blood flow has to work under conditions of dominance of viscous forces. Analytical models have shown that peristaltic pumping can generate significant flow under these conditions [24,29], whereas traditional (straight) Liebau-effect pumps fail to work or do not work very efficient [24,93,114,115]. Our present data show that kinking and torsion can significantly improve the efficiency of Liebau-effect pumps for pumping of low viscous (distilled water) as well as high viscous fluids (1:1 mixture of water/corn syrup). It, therefore, may be tempting to interpret our findings as evidence disproving the above-mentioned argument against the postulated action of the Liebau effect in valveless heart tubes. We have to note, however, that downscaling of our pump models, via usage of high viscous working fluid, did not fully reach the scales of microvascular flows within vertebrate embryonic heart tubes. The Reynolds and Womersley numbers encountered in our experiments of high viscous fluid pumping were higher than 1 (Table 1 and Table 2). The values correspond to the scales of the smallest (terminal) branches of the arterial and venous vascular trees [104] and also to the scales of the valveless heart tubes of some basal chordates (tunicates) [24,25]. They do not correspond, however, to the scales of vertebrate embryonic heart tubes, which are commonly characterized by Reynolds and Womersley numbers less than 1 [23,24]. In view of this fact, we cannot be sure that our data can be correlated to the situation of embryonic heart tubes. Thus, if we want to gain full support of our hypothesis, future studies are needed to specifically clarify (1) whether kinking and torsion may make Liebau-effect pumps suitable for pumping at very low Reynolds (Re ≤ 1) and Womersley (Wo ≤ 1) numbers; and (2) whether kinking may also increase the efficiency of further valveless pumping mechanisms such as peristalsis. With respect to the latter question, we should note that several biological conduits that move body fluids by propulsive peristaltic contractions (e.g., esophagus, ureter, lymphatics, peristaltic veins) possess sphincters or valve-like anatomical structures [121,122,124,125], suggesting that such structures may improve the function of peristaltic pumps. In view of the postulated valve-like or sphincter-like action of kinked portions of blood vessels (see above), this fact led us speculate that kinking not only might improve the pumping efficiency of Liebau-effect pumps but also of peristaltic pumps.

### 3.7. Technical Relevance

We should finally note that our present data might stimulate not only a rethinking about form-function relationships of blood vessels and valveless biological pumps. They might also lead to an improvement of the functional design of technical Liebau-effect pumps. With respect to the latter point, we should emphasize that the modifications of the pump design (kinking and torsion) leading to the improvement of the efficiency of our Liebau-effect pump were inspired by form features normally found in valveless biological pumps (tubular hearts of vertebrate embryos and tunicates). Thus, our present study nicely demonstrates the well-known fact that biologically inspired design can improve the function of man-made technical devices [126]. With respect to Liebau-effect pumps, our study is not the first to show that bio-inspired modifications of the traditional pump design can improve their function. The traditional design of man-made Liebau-effect pumps is that of a straight and uniform cylindrical conduit with a relatively thin wall consisting of a homogeneous elastic material. Inspired by the thick and multi-layered wall structure of vertebrate embryonic heart tubes, Loumes and co-workers [98] modified the traditional design of man-made Liebau-effect pumps by the addition of a thick gelatinous internal layer to the wall of the pump. Compared to thin-walled pumps, only small excitations were needed to generate significant flow rates in the thick-walled, multi-layered variant. The beneficial effect of pump wall thickening was recently confirmed by a study conducted by Koslovsky and co-workers [27]. This group furthermore reported that the pumping efficiency of a Liebau-effect pump could be increased by another bio-inspired modification of its traditional pump design, namely the creation of a chamber (“atrial chamber”) at the site of compression. Interestingly, and corresponding to our present findings, this group also reported that the beneficial effects of their pump design modifications were especially significant at the scales of small blood vessels. Based on these findings, they concluded that the efficiency of the valveless pumping in the tubular heart is increased due to its unique anatomy and the range of working parameters.

## Figures and Tables

**Table 1:** Womersley numbers encountered in the pumping experiments.

	0.5 Hz	1.0 Hz	1.5 Hz	2.0 Hz	2.5 Hz	3.0 Hz
Dist. Water	4.47	6.32	7.74	8.94	9.99	10.95
Water-Syrup	1.36	1. 93	2.36	2.73	3.05	3.34

**Table 2:** Reynolds numbers encountered in the pumping experiments.

	0.5 Hz	1.0 Hz	1.5 Hz	2.0 Hz	2.5 Hz	3.0 Hz
Dist. Water straight tube	52.08	122.13	147.02	148.80	132.05	194.14
Dist. Water looped tube	102.43	98.20	132.85	152.75	133.68	214.88
Water-Syrup straight tube	1.81	3. 29	3.92	4.19	5.41	5.33
Water-Syrup looped tube	2.68	4.33	5.73	7.22	8.66	8.89
